# Observations and Models of Highly Intermittent Phytoplankton Distributions

**DOI:** 10.1371/journal.pone.0094797

**Published:** 2014-05-02

**Authors:** Sandip Mandal, Christopher Locke, Mamoru Tanaka, Hidekatsu Yamazaki

**Affiliations:** Department of Ocean Sciences, Tokyo University of Marine Science and Technology, Minato-ku, Tokyo, Japan; University of Shiga Prefecture, Japan

## Abstract

The measurement of phytoplankton distributions in ocean ecosystems provides the basis for elucidating the influences of physical processes on plankton dynamics. Technological advances allow for measurement of phytoplankton data to greater resolution, displaying high spatial variability. In conventional mathematical models, the mean value of the measured variable is approximated to compare with the model output, which may misinterpret the reality of planktonic ecosystems, especially at the microscale level. To consider intermittency of variables, in this work, a new modelling approach to the planktonic ecosystem is applied, called the closure approach. Using this approach for a simple nutrient-phytoplankton model, we have shown how consideration of the fluctuating parts of model variables can affect system dynamics. Also, we have found a critical value of variance of overall fluctuating terms below which the conventional non-closure model and the mean value from the closure model exhibit the same result. This analysis gives an idea about the importance of the fluctuating parts of model variables and about when to use the closure approach. Comparisons of plot of mean versus standard deviation of phytoplankton at different depths, obtained using this new approach with real observations, give this approach good conformity.

## Introduction

Seasonal variation of nutrient and dynamics of plankton in aquatic systems have been studied by many researchers during the last few decades, and mathematical models have been used to better understand these systems. Ecological models have evolved from simple two-component systems to highly complex multi-component systems [1]–[15]. The complexity of a model increases significantly when coupled with a physical system to make a biophysical model [16]–[23]. However, these models study the system dynamics in mesoscale or in larger scale but are not aimed at explaining the dynamics in microscale.

Interactions of phytoplankton, other organisms and biogenic particles in turbulent flows drive plankton dynamics and cycling of nutrients, with influences expected to be accumulative across multiple scales [24]. As technology improves and measurements of phytoplankton distributions are done with greater resolution, properly interpreting *in situ* spatial patterns becomes increasingly important for developing our knowledge of the mechanisms that create and support microscale structure and ecology of marine ecosystems [25], [26]. Phytoplankton measurements of microscale distributions have recently been studied using a variety of high-resolution instruments, such as water sampling devices [27], microstructure profiling fluorometers [26], [28]–[30], fluorescence imaging systems [31], [32] and holography systems [33]–[35]. Various fluorescence-based measurement systems have consistently shown increasing levels of fluorescent intermittency as sample volume size is decreased [26], [28], [30], [31], which can be attributed to the preferential detection of larger individual cells, chains and aggregates [36]. Furthermore, efforts to understand the mechanisms driving the high levels of observed intermittency using conventional (Gaussian) models, such as spectral analysis [37], appear to be limited by the discrete nature of phytoplankton distributions at small scales [26], [36], [38]. Highly resolved profiles of phytoplankton concentration and fluorescence would give better predictions of model parameters and improve understanding of the dynamics of phytoplankton [22]. These high-resolution instruments advance our understanding of the influence of physical processes on phytoplankton dynamics from mesoscale down to microscale. Therefore, an analysis of small-scale vertical structure in phytoplankton is required to understand the ecosystem behaviour more precisely.

### Rationale for the development of a new ecosystem model

Interactions between different biotic and abiotic factors [39], [40], at the microscale, generate spatial variability in the distribution of phytoplankton [31], [41]. Profiles of *in vivo* chlorophyll fluorescence are commonly used to infer the distribution of phytoplankton biomass.

**Figure 1:**
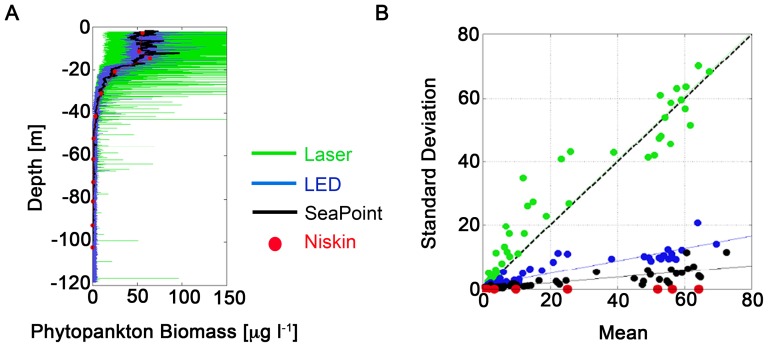
Examples of fluorescence microstructure measurements by Niskin bottle (red), Seapoint (black), LED sensor (blue) and laser sensor (green). (A) Plot of data at different depths from the ocean surface at the mouth of Tokyo Bay, Japan, on June 18, 2011, and (B) mean and standard deviation of chlorophyll signals by four sampling methods. Data from Seapoint, LED sensor and laser sensor were scaled to data obtained by Niskin bottle, which gives the actual concentration of phytoplankton.

Figure 1A shows an observation of chlorophyll profiles (phytoplankton) collected at the mouth of Tokyo Bay, Japan, in June 2011, up to 120-m depth from the ocean surface using four sampling methods: Niskin bottle, Seapoint fluorometer, light emitting diode (LED) sensor and laser sensor (Dataset S1). Descriptions of the instruments and sampling methods are described briefly in the next section. This survey was carried out with permission of the Japan Coast Guard (http://www.kaiho.mlit.go.jp/e/index_e.htm).

Comparison of fluorescence profiles using these sensors shows that the extent of phytoplankton spatial variability becomes increasingly intermittent when measured with increased resolution. However, 1-m averaged fluorescence levels (spatial mean) are consistent for all four devices. Spatial mean of the collected chlorophyll data and the corresponding standard deviations in all four methods are plotted in Figure 1B. For Niskin samples, the standard deviation of phytoplankton is zero since only a single number for average chlorophyll from the water sample is available. High-resolution data show high spatial variability of phytoplankton near the ocean surface and that variability and mean decrease with depth. In theoretical studies of phytoplankton dynamics, especially in formulating ecological models, almost all modellers focus on the large-scale dynamics of phytoplankton and the mean value of the collected data, not considering the associated small-scale spatial variability.

Our observed data suggest that considering the fluctuating part of each component of the system as a new variable is important, particularly with high-resolution data. This paper aims to study the effect of this variability on the model output and to compare this effect with real observations.

## Materials and Methods

### Microstructure data collection

Four sampling methods, namely Niskin bottle, Seapoint fluorometer, light emitting diode (LED) sensor and laser sensor, were used for data collection. The Niskin bottle and the Seapoint fluorometer were mounted on a CTD cage, which was tethered to a winch cable. Swaying with the motion of ship, the CTD cage generated a disturbed water column. The Niskin bottle is a discrete sampling device, which measures the “averaged” ecosystem characteristics by collecting water samples. The sampling volume of Seapoint is 0.34 mL, but the motion of the CTD cage and the configuration of the probe did not allow for sampling from undisturbed water. In contrast, the LED and the laser sensor were mounted on a free-fall microstructure profiler, TurboMAP-L [26]; thus, the sampling was made from essentially undisturbed water.

The minimum vertical resolution for the LED sensor is about 2 cm and is about 2 mm for the laser sensor [26]. Microscale spatial variability observed by the LED sensor varied by less than 2-fold across centimetre to metre scales. In the laser sensor, the microscale spatial variability varied by between 2- and 10-fold across millimetre-size peaks. The reduced sampling volume of the laser probe (32 µL), compared with that of the LED probe (4 mL), allows for measurement of the fluorescence field with increased spatial resolution.

High-resolution microstructure fluorescence data were collected in several locations in Tokyo Bay at different time periods using a laser probe mounted in a TurboMAP-L [26] (Dataset S1). TurboMAP-L is a free-falling microstructure profiler that captures physical and biological conditions. The instrument length is 2 m, the weight is 30 kg in air, the diameter is 0.12 m, and the withstanding pressure is 500 dbar (∼500 m). The captured data were transmitted in real time to the shipboard PC via a 5 mm Kevlar tether attached to the end of the instrument. TurboMAP-L was deployed and freefell into the sea with profiling speeds of 0.50–0.80 ms^−1^. After reaching the target depth, the instrument was recovered by taking up the tether using a winch exclusive to TurboMAP-L.

The sensors attached to TurboMAP-L are a laser fluorescence probe (digitized at 256 Hz), a light emitting diode (LED) fluorescence/turbidity probe (256 Hz), two turbulent shear probes (512 Hz), a FP07 fast temperature probe (512 Hz), XYZ 3-dimensional accelerometers (64–256 Hz) and CTD (Conductivity, Temperature and Depth; 64 Hz). These sensors were mounted forward of the rounded nose to ensure the measurement of undisturbed fields.

In May 2011, the experiments were conducted at three locations in Tokyo Bay: (a) the mouth of the bay (depth ∼500 m), (b) inside the bay (depth ∼50 m) and (c) the mouth of the Ara River (depth ∼10 m). In these locations the microstructure data were collected from TurboMAP-L to a depth of ∼200 m, ∼50 m and ∼10 m. Similarly the fluorescence data were collected at a particular place inside the bay (depth ∼50 m) but in three different seasons, specifically during the months: (a) December 2006, (b) September 2007 and (c) February 2008. Normally arbitrary units of fluorescence are calibrated to give estimates of biomass in µg l^−1^. Permission for these surveys was received from the Japan Coast Guard.

### Mathematical model

Aiming to develop the methodology of a new ocean ecosystem model, at the initial stage, we concentrate only on nutrient (*N*) and phytoplankton (*P*) as model variables and choose simple form for transfer functions [5] (Section S1 in Text S1).

#### Simple NP model

The time evolution equations for an NP system can be written as:
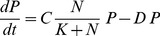
(1)

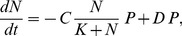
(2)where parameters are described in Table 1. The sum of *N* and *P* variables is a conserved quantity, i.e. *N*+*P* = *A* (Constant). This reduces the number of equations into one,
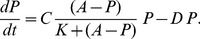
(3)


**Table 1:** Definition of different quantities used in the model and their dimensions.

Quantity	Definition	Dimension	Scaling factor	Dimensionless quantity
*A*	Sum of nitrate	µg N l^−1^	-	-
*B*	Variance of sum of fluctuating components	(µg N l^−1^)^2^	*B/A^2^*	*B*
*C*	Maximum growth rate of phytoplankton	day^−1^	-	-
*K*	Nutrient uptake half-saturation constant	µg N l^−1^	*K/A*	*K*
*D*	Phytoplankton death rate	day^−1^	*D/C*	*E*
*P_0_*	Mean phytoplankton	µg N l^−1^	*P_0_/A*	*p_0_*
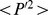	Variance for phytoplankton	(µg N l^−1^)^2^	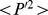 /*B*	*X*
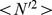	Variance for nutrient	(µg N l^−1^)^2^	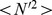 /*B*	*Y*
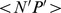	Covariance	(µg N l^−1^)^2^	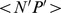 /*B*	*Z*
*t*	Time	day	*t C*	*Τ*

Studies of microscale phytoplankton distributions using high-resolution profiling fluorometers have revealed the ubiquity of fluctuating millimetre- to centimetre-sized fluorescence peaks across a range of marine systems, which also implies the local values of fluorescence are highly fluctuating [26], [38] in space. Since the amount of nutrient in the system is interrelated to the biomass of phytoplankton, the nutrient would be expected to be fluctuating in space for a close system. But this spatial variability is not considered in the simple non-closure model as described in equations (1), (2) or (3).

Therefore, considering our model variables as functions of time (*t*) and space (*r*), and decomposing each variable into mean and fluctuating component, we get 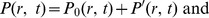
(4)

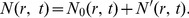
(5)



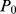
 and 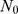 are spatial mean values of the phytoplankton and nutrient respectively, and 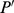 and 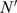 are their fluctuating components corresponding to each mean value. Note that at a particular time point, the space-average of the fluctuating components is zero (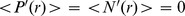), while temporal average can be nonzero, which also implies 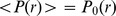 and 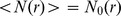. This consideration is based upon our observation of Figure 1, where we see that 1-m averaged fluorescence levels (spatial mean) for all four instruments coincide. Now we are interested in seeing the temporal variation of mean and fluctuating component of *P* and *N* variables using the closure approach, which is widely used in the study of turbulence.

#### Closure model

Putting (4) and (5) into equations (1–2) and applying the Reynolds averaging method in space (details in Section S2 in Text S1), we get the following set of equations for the temporal variation of 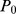, 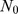, 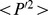, 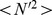 and 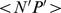: 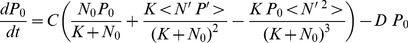
(6)

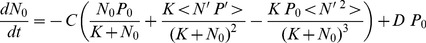
(7)

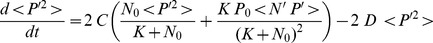
(8)

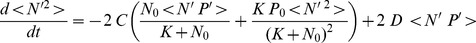
(9)

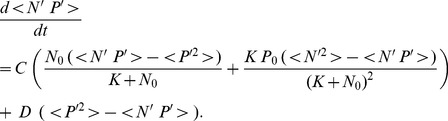
(10)


In the formulation of these equations we assume that *N* and *P* variables follow a joint lognormal probability distribution function, which forces the third and all higher odd order fluctuating terms to vanish. We also ignored the fourth and higher order terms in this analysis to achieve simple closure. The first two equations give the time evolution of mean terms, the next two equations give the time evolution of variance terms, and the last equation represents the evolution of the covariance term.

Note that while the sum of 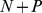
remains constant, in this case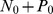 and 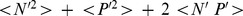, both are temporally conserved quantities. Therefore, defining 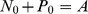 and 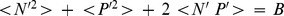, the above five equations can be reduced to three equations that lie in five-dimensional parameter spaces. By appropriately rescaling the equations with *A* and *B*, this dependency is reduced to three dimensionless parameters. The scaling factors and dimensionless parameters are given in Table 1, and the scaled equations can be written as follows:
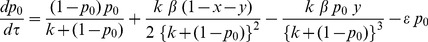
(11)

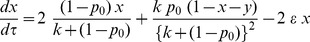
(12)

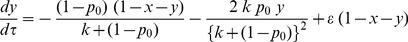
(13)with 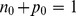 and 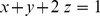.

The values of the scaled variables *n_0_* and *p_0_*, corresponding to the variables *N_0_* and *P_0_* respectively, lie between 0 and 1. Similarly, the values of x and y lie between 0 and 1 if the term z, which is associated with the covariance of the fluctuating components, 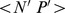, is positive. For negative covariance, the values of x and y can exceed 1. Now we define the normalized sum of variance and covariance as follows:
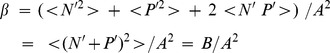
(14)where *B* is the variance of the sum of 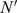 and 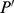, and therefore, *β* value actually reflects the overall strength of the fluctuating components and modifies the model dynamics. Other dimensionless variables and parameters of the model equations (11–13) are described in Table 1.

Similarly, the non-closure equation (3) can be reduced to the following form:
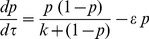
(15)


Note that for *β* = 0, equation (11) of the closure model reduces to equation (15), which corresponds to the non-closure model.

We have found six steady state solutions (p_0_*, x*, y*) for the set of closure equations (11–13), among which two are not ecologically feasible as these are complex for any realistic set of parameter values (Sections S3 and S4 in Text S1). Two other solutions lie at the boundary, and the remaining two are interior steady states. Depending on the sign of the covariance term (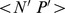), between these two, only one interior steady state exists for the system. Also, from stability analysis, we have seen that the interior steady state is stable when the covariance term is positive (Section S5 in Text S1). Therefore, all analysis was done emphasising this stable steady state solution.

#### Parameter values

After reducing the equations into dimensionless variables and parameters, the influences of only three parameters on the model have to be studied. Here, the model under study is a general one and does not describe any specific ecological situation. Therefore, for numerical simulation, the assigned parameter values are adopted from previous studies [2], [19], [42]–[44]. To find the domain of parameter space for this model, we have studied a wide range of parameter values around these reported values.

Maximum growth rate of phytoplankton (*C*) varies with the intensity of light, although at the first stage of this work *C* is considered to be 2 day^−1^ [2], [19], while 2 µg N l^−1^ is chosen as the value of *A* [2]. Different literature shows that the death rate (*D*) of phytoplankton varies from 0.07 day^−1^ to 0.2 day^−1^ [2], [19], [42]. For the neritic zone, MacIsaac and Dugdale [43] and Eppley *et al*. [44] had considered the value for the half-saturation constant of phytoplankton nutrient uptake *K* to be 1 µg N l^−1^, whereas Edwards *et al*. [19] had used 1.4 µg N l^−1^ as this parameter value.

For our numerical simulation, we have varied the dimensionless parameter *ε* ( = *D/C*) from 0.025 to 0.99 and k ( = *K/A*) from 0.05 to 2.5, covering the reported values range. All the reported parameter values, their ranges and the corresponding dimensionless values are summarized in Table 2. The domains of parameter values, which the system of equations (11–13) shows to have stable feasible solutions, are shown in Figures S1, S2, S3, S4, S5 and S6 (See Sections S4 and S5 in Text S1).

**Table 2:** Parameter values and ranges for dimensional and dimensionless systems.

Quantity	Reported value with references	Corresponding dimensionless quantity	Corresponding dimensionless value	Range of parameter values studied in this article
*A*	2 µg N l^−1^ [2]	-	-	-
*C*	2 day^−1^ [2], [19] (varies with light intensity)	-	-	-
*K*	1 µg N l^−1^ [43], [44], 1.4 µg N l^−1^ [19]	*k*	0.5–0.7	0.05–2.5
*D*	0.07 day^−1^ to 0.2 day^−1^ [2], [19], [42]	*ε*	0.035-0.1 (for C = 2 day^−1^); 0.35-1.0 (for C = 0.2 day^−1^)	0.025–0.99

## Results and Discussion

This article attempts to capture the spatial variability of different constituents (nutrient and phytoplankton) of an ocean ecosystem through mathematical models and to understand the contribution of the fluctuation part of model variables in the system dynamics. Here the dimensionless parameter *β* actually represents the ratio of overall variance of the fluctuating part to the square of total nutrient contained in the system, i.e., 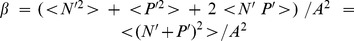. We have found a critical value of *β* below which both closure model and non-closure model give the same steady state level.

### Critical parameter value

**Figure 2:**
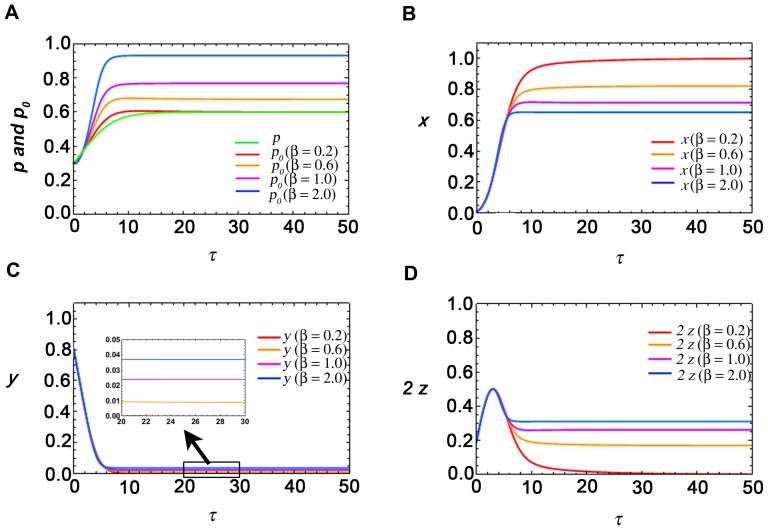
Time variation of variables p_0_, x, y and 2z of the closure model. Plots of these variables are shown in Figures A, B, C and D respectively for β = 0.2, 0.6, 1.0 and 2.0. Variation of p of the non-closure model is simultaneously depicted in Figure A. The constant parameter values for this simulation are k = 0.6 and ε = 0.4, for which β^*^ = 0.36.

For a certain choice of parameter values, from our studied parameter domain, Figure 2 shows the time dynamics of the variables *p_0_*, *x*, *y* and *z*, which correspond to mean of phytoplankton, variance of phytoplankton, variance of nutrient and covariance of *x* and *y*. In these plots, *k* and *ε* values are kept constant at 0.6 and 0.4 respectively (chosen from the realistic parameter domain in Table 2), and for the closure model four *β* values are chosen, *β* = 0.2, 0.6, 1.0 and 2.0. Time dynamics corresponding to the variable phytoplankton for both the closure and the non-closure model are simultaneously plotted in Figure 2A. Figures 2A–D show that the values of *p_0_*, *y* and z increase as the *β* value increases, whereas *x* decreases with the increase of *β*. According to Figures 2A and B, the variance of phytoplankton (*x*) can be greater or less than the mean value (*p_0_*) depending on the value of *β*. Figure 2A shows that for *β* = 0.2 the steady state level of the closure model coincides with the steady state level of the non-closure model although their transient dynamics are different. But as *β* increases, beyond some critical value of *β* = β*, the steady state levels of phytoplankton in these models do not coincide, and the difference increases as *β* increases (also see Section S6 in Text S1, Figures S7 and S8). This gives an idea about the limit of the parameter *β* above which the closure approach is appropriate to use and below which the non-closure model agrees with the results from the closure model.

This critical parameter value of *β* can be obtained by equating the nonzero steady state value for *p_0_* of the closure model and *p* for the non-closure model (see Section S3 in Text S1). Thus, at *β* = β^*^ we have the following expression for β^*^: 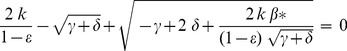
(16)with, 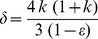
(17)

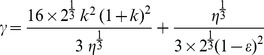
(18)and
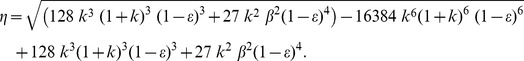
(19)


**Figure 3:**
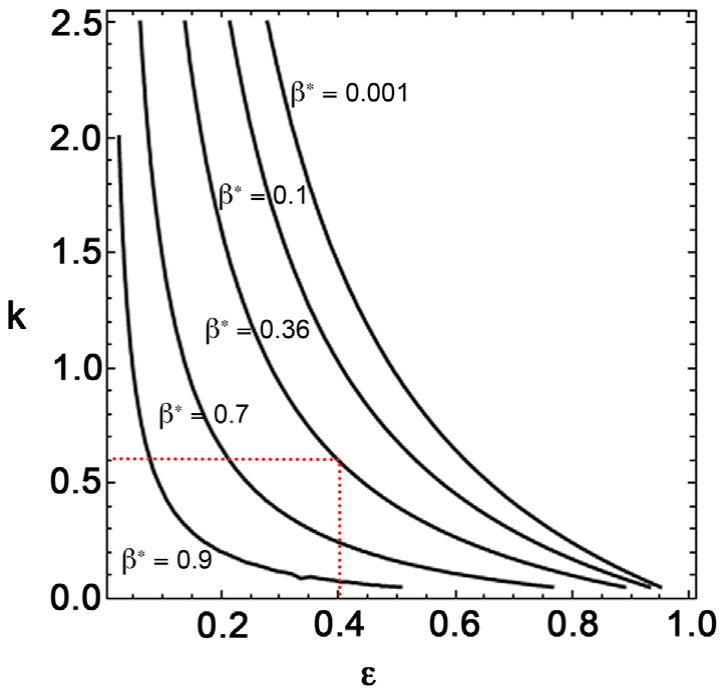
Plot of critical value of β ( = β*), with the variation of ε for k. The red dotted line indicates the β* value corresponding to specific k and ε values.

Figure 3 shows the variation of the critical parameter β^*^ with the variation of *k* and *ε*. For a particular value of *ε* and *k*, β^*^ value corresponds to the maximum value of *β* up to which the non-closure model and the closure model reach the same steady state value for phytoplankton. However, beyond that value, the closure model shows a different result. For example, from equation (16) we can determine the β^*^ value as 0.36 for *k* = 0.6 and *ε* = 0.4, which clearly justifies our numerical results above as shown in Figure 2A: for *β* = 0.2 (<0.36) the closure model approaches the non-closure model, but for *β* = 0.6, 1.0 and 2.0 (>0.36) the closure model gives a different result from the non-closure model.

From the same figure it is also observed that as the value of the parameter *ε* decreases, the β^*^ value increases. This means that as the death rate of phytoplankton (*D*) decreases or as the growth rate of phytoplankton (*C*) increases, the critical value of *β* increases. Similarly, as the value of *k* ( = *K*/*A*) decreases, the critical value of *β* increases. These phenomena imply that the closure model has a more important role where the growth rate of phytoplankton is less with respect to death rate and half-saturation constant (*K*) is high with respect to total nutrient content (*A*) of the system.

### Coefficient of variation (CV) of phytoplankton in closure model

In real observations we can never measure data with full certainty. To standardize our measurement, we normally consider the mean value of several collections of that data along with its standard deviation. In this situation, a statistical parameter, which becomes important, is the coefficient of variation (CV). This is a normalized measure of variability of a distribution and is defined as the ratio of standard deviation to the mean of that variable.

Because the closure model considers the fluctuation of variables, one can expect to determine the coefficient of variation in this model. To do this, we first recall our model variables *P_0_*, *N_0_*, 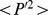 and 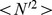. *P_0_* and *N_0_* represent the mean of phytoplankton and nutrient respectively.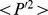 and 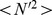 represent the variance of fluctuating component corresponding to those variables. The square root of the variance is a measure of standard deviation.

From Figure 2 we have seen that for a particular value of *k* and *ε*, all the variables reach a steady state level after some time period. This level varies as *β* varies. We have also seen from Figures 2A and B that the variance of the fluctuating component of phytoplankton (*x*) can be greater or less than the mean value of phytoplankton (*p_0_*) depending on the value of *β*. Therefore, the coefficient of variation for phytoplankton can be greater or less than one, depending on the parameter values.

**Figure 4:**
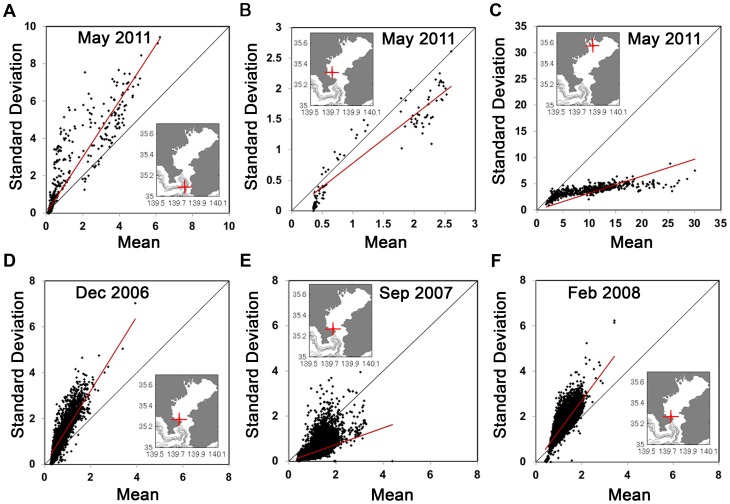
Observations of phytoplankton data in Tokyo Bay. Figures (A, B, C) show mean and standard deviation of fluorescence microstructure data obtained from different locations in Tokyo Bay in May 2011. (D, E, F) show data obtained from the same location in Tokyo Bay but at different time periods. Slope of the trend line passing through the origin of these data sets (red line) gives the effective value of the coefficient of variation at that location. Inset figures show the data collection locations.

Microscale fluorescence measurements at different locations in Tokyo Bay and at different time periods are shown in Figure 4 (Dataset S1). Permission for these surveys was received from the Japan Coast Guard. Scattered points represent the mean and standard deviation of the fluorescence measurements over one-meter vertical intervals. Figures 4A–C show that the effective coefficient of variation at the mouth of the bay, inside the bay and at the mouth of the Ara River to varied from 1.50, 0.78 and 0.32 respectively in May 2011. These spatial structures imply that at the same period but at different locations the coefficient of variation of phytoplankton can be different. This is because of a combination of the variation of physical processes and the community structure of phytoplankton at different locations that influences the plankton ecology. Figures 4D–F demonstrate the fluorescence data at a particular place inside the bay (depth ∼50 m) but in three different seasons. In December 2006, the coefficient of variation was 1.61, which becomes 0.37 (<1) in September 2007. Again, in February 2008, the value of the coefficient of variation increases to 1.36 (>1). Therefore, at a particular location the coefficient of variation changes seasonally with the seasonal change of physical dynamics as well as with the change in the community structure of phytoplankton.

Now we will see how the newly developed closure model can capture these changes of the coefficient of variation by changing different parameters. In a particular study area of ocean, when considering the abundance of a particular species of phytoplankton, we can assume that the average death rate (*D*) and half-saturation constant (*K*) of phytoplankton are constant. As an example, let us consider the values of *D* and *K* as 0.135 day^−1^ and 1.2 µg N l^−1^ respectively, which are actually the average values of the reported cases (Table 2). We also assume the total nutrient of the system to be constant, and during the first step we set its value to be 2 µg N l^−1^ (Table 2). Therefore, among three main parameters (*C*, *K* and *D*) of the original model, two (*K* and *D*) are constant over time and space (here space actually represents the depth). The maximum growth rate, *C*, depends on the intensity of light and varies with depth. As the intensity of light decreases with the depth from the sea surface, we assume that the maximum growth rate of phytoplankton decreases with depth.

**Figure 5:**
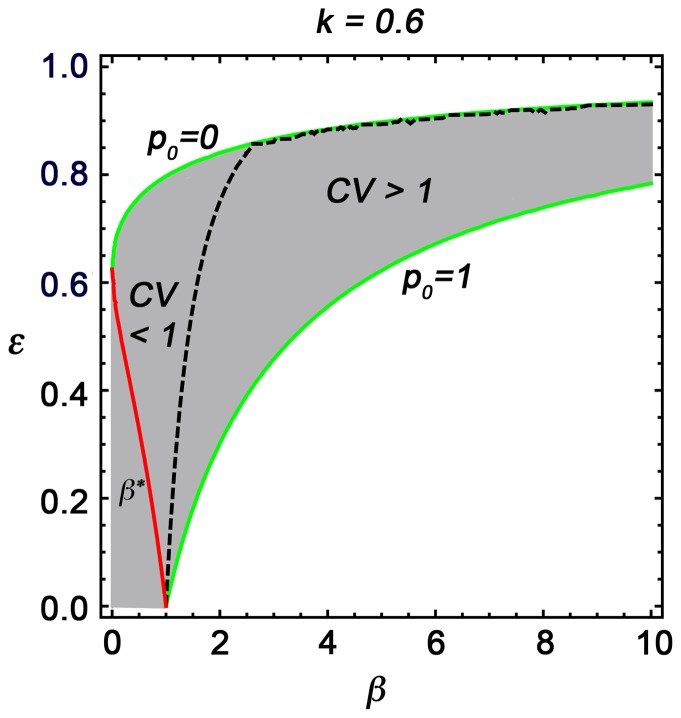
Domain of parameters for the value of coefficient of variation (CV) of phytoplankton. The grey region is the domain of parameters in the (ε-β) parameter space for p_0_ to lie between 0 and 1. Green lines are the boundary values corresponding to the mean of phytoplankton (p_0_). The dashed line divides the parameter domain for CV<1 and CV>1.

To study our system of equations (11–13) we must look after the values of the dimensionless parameters. The value of *k* is 0.6 as *k* = *K*/*A*, and the value of *ε = D/C = 0.135/C*. Considering the maximum value of *C* at the surface as 2 day^−1^ gives the minimum value of *ε* = 0.067, and according to our model formulation, the maximum value of *ε* is <1. Therefore, the value of *ε*, which is the ratio of phytoplankton death rate to the maximum growth rate of phytoplankton, at a specific depth, is the minimum at the ocean surface, and its value increases as depth increases. Figure 5 shows the domain of parameters in the *ε-β* parameter space for *p_0_* to lie between zero and one. The red line indicates the value of β* below which the mean value of phytoplankton for the non-closure and the closure model coincide. The dashed curve in the grey shaded region divides the entire parameter domain of the closure model into two sections: on one side the coefficient of variation (CV) for phytoplankton is less than one and on the other, is greater than one. We have also observed that as the total nutrient (*A*) of the system decreases, the domain of parameter in *ε-β* space for CV<1 decreases and the parameter domain for CV>1 increases (see Section S7 in Text S1, Figure S9). Now we will see the variation of mean and standard deviation for phytoplankton at different depths within this described parameter domain.

**Figure 6:**
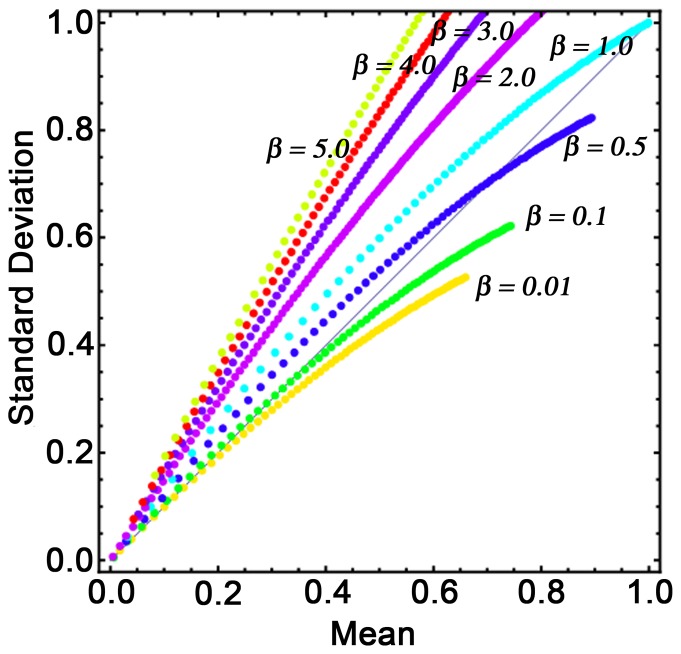
Plot of mean versus standard deviation (SD) of phytoplankton at different depths for eight different β values. Each point corresponds to the mean and SD of phytoplankton at a particular depth. Both axes are normalized by the value of A ( = 2 µg N l^−1^). Here the depth profile is obtained by changing the parameter value C, which decreases as depth increases, and the other two parameters K and D are kept constant at 1.2 µg N l^−1^ and 0.135 day^−1^.

Figure 6 shows the normalized mean (*P_0_/A*) and standard deviation (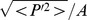) corresponding to phytoplankton for different *β* values. This is obtained by giving back the dimensions of the variables of the system of equations (11–13). Each point in this plot represents the steady state value of mean and standard deviation at a specific value of *ε* ( = *D/C*). The value of *ε* increases with depth as the growth rate of phytoplankton (*C*) decreases with depth due to light attenuation, even if the average death rate of phytoplankton (*D*) remains constant. Therefore, for a particular *β* value, the changes of *ε* value give us the depth profile of phytoplankton from our model simulation. Eight *β* values (*β* = 0.01, 0.1, 0.5, 1.0, 2.0, 3.0, 4.0 and 5.0) are chosen to show these results, and for a particular *β* value, the ranges of *ε* values are determined from the parameter domain of Figure 5. As an example, for *β* = 3.0, the value of *ε* can vary from 0.46 to 0.86. From these plots, we observe that as the depth increases, the mean value of phytoplankton as well as its standard deviation decreases, which implies that the mean of phytoplankton and its spatial variability decrease with depth. This model result is justifiable, as we see similar behaviour in small-scale observation of phytoplankton data, depicted in Figure 4.

In real observations we see the coefficient of variation vary from less than one to greater than one (Figure 4). In Figure 6, the diagonal line characterizes the points where the mean and standard deviation are equal, indicating that the coefficient of variation is equal to one along this line. From our model results we also see the coefficient of variation vary on both sides of the diagonal line depending on the value of the parameter *β*. This figure also shows that as *β* increases, the coefficient of variation increases. If the total nutrient of the system, *A*, is conserved, then the change of *β* implies the change of fluctuating components of the system. This is because *β* = *B*/*A* ^2^, and *B* represents the variance of overall fluctuating components. Therefore, according to our analysis, as the variance of the overall fluctuating components of the system increases, the coefficient of variation increases.

We have also observed that the parameter *β* is more sensitive to the coefficient of variation when the total nutrient (*A*) of the system is high. These mathematical observations imply that in a particular area of ocean with high total nutrient, spatial variation will be low for low *β* value and therefore, the coefficient of variation will be low. This may be the reason for low coefficient of variation at the mouth of the Ara River (Figure 4C). Low values of CV may imply that phytoplankton species with less sticky material may dominate the community structure. As the seasons change, the ocean dynamics change, which causes the spatial variability of the phytoplankton species to vary. In the closure model this change is observed by changing the parameter *β*. Therefore, Figures 4D, E, and F can be explained by stating that the *β* value changes seasonally and causes the coefficient of variation to change seasonally.

### Estimation of parameter values

In our closure model the phytoplankton death rate *D* is a measurable quantity and was estimated by Lehman *et al*. [42], Franks *et al*. [2], Edwards *et al*. [19] and many other researchers. Similarly, the value of half-saturation constant, *K*, the maximum growth rate of phytoplankton, *C*, and the total nutrient of the system, *A*, were also measured by Franks *et al.* [2], Edwards *et al.* [19], MacIsaac and Dugdale [43] and Eppley *et al.* [44]. On the other hand, in our model, *B* is a conserved quantity that essentially measures the sum of variance and covariance terms (or equivalently the variance of total fluctuating components) and is not easily measurable. One can guess the value of this quantity by estimating the parameter *β*, as *B* = *β×A* ^2^. This parameter can be estimated by using *β*-value, at which the coefficient of variations ( =  Standard deviation/Mean) of the observation agrees with the coefficient of variation of the model result (Figure 6). Therefore, the closure model can also be used to estimate this unknown parameter of the system.

## Conclusions

Mathematical modelling is a pillar of theoretical ecology research. Aimed at addressing various questions, several models have been developed over the decades and range from very simple to the highly complex. Predictive models are always validated or compared with measured data sets. However, sometimes a problem occurs where the measured variables are highly intermittent. Technological advances allow for measurement of phytoplankton data to greater resolution in oceanic ecosystems. High-resolution data, which displays high spatial variability, are useful for understanding the influences of physical processes on phytoplankton dynamics. In conventional methods, the mean value of the measured variable is approximated to compare with the model output. This approximation may misrepresent the reality of planktonic ecosystems, especially at microscale level. To consider spatial variability of variables, in this article, we have applied a new modelling approach, the closure approach, to the planktonic ecosystem. The closure approach has been widely used for numerical models in turbulence research. In this approach, the variables are separated into mean and fluctuating parts, and the fluctuating parts are considered new variables of the system. By taking the spatial average of the governing equations, we get the closure equations, which are then solved to see the effect of fluctuation on the mean variables.

To justify this closure approach, as a first step we have considered the simple two-component NP system. After dividing each variable into mean and fluctuating parts, using the closure approach technique we have obtained five evolution equations among which two belong to the mean value of the variables, two correspond to the variance terms, and the last corresponds to the covariance term. Analysing this set of equations, first we found the domain of parameters and conditions for stability of the system (see Sections S4 and S5 in Text S1). We have observed that the increase of variance of overall spatial fluctuation (*β*-value) increases the stability zone of the system (). Then we determined the critical value of fluctuation below which the non-closure modelling approach is sufficient to describe the dynamics of the system but above which the system attains a new steady state, which shows why the closure approach is necessary above this critical limit.

As this modelling approach handles variance and covariance terms, simulated results of this model are easily comparable with observed coefficients of variation. Depending on the variance of overall fluctuation (*β*-value), the system may have coefficients of variation greater or less than one. We have observed from our simulation that for high fluctuation, the coefficient of variation is higher, and we have also found a parameter domain (*ε-β* space) where the coefficient of variation is greater or less than one. By comparing model results with observed data sets, physically unmeasured parameter values, such as variance of overall fluctuation, can also be estimated.

Although the whole analysis is performed on a simple NP model, the results of this new approach show some beautiful features that we also observe in the real oceanic ecosystem. This novel technique for planktonic ecosystem modelling can be used for a system where the observed data sets are highly intermittent and normally difficult to compare with conventional non-closure models. Also, this method gives a general idea about the contribution of fluctuating components of the variables, which are generated by various biotic and abiotic factors of the system. In the next step of our work, we plan to incorporate physical dynamics, extend model variables in a more realistic way using this methodology and observe the effect of intermittency on the variables. How these fluctuating terms will change the results of global climate models when the time integration is extended over a few decades or more is not clear, but the results would be different from current predictions using these methods. The future of the climate is still unknown.

## Supporting Information

Figure S1 **Domain of parameter values for which the equilibrium point E_2_ exists.** Grey shaded regions indicate the domain for (A) β = 0.1, (B) β = 0.5, (C) β = 1.0, (D) β = 1.5, (E) β = 4.0 and (F) β = 10.0. Green, blue and red lines are the boundary values corresponding to the mean of phytoplankton (p_0_), variance of fluctuating component associated with phytoplankton (x) and nutrient (y) respectively.
(TIF)

Figure S2 **Domain of parameter values for which the equilibrium point E_3_ exists.** Grey shaded regions indicate the domain for (A) β = 0.1, (B) β = 0.5, (C) β = 1.0, (D) β = 1.5, (E) β = 4.0 and (F) β = 10.0. Green, blue and red lines are the boundary values corresponding to the mean of phytoplankton (p_0_), variance of fluctuating component associated to phytoplankton (x) and nutrient (y) respectively.
(TIF)

Figure S3 **Stability zone of the equilibrium point E_0_.** Two-parameter bifurcation diagram to show the stability zone for (A) β = 0.1, (B) β = 0.5, (C) β = 1.0, (D) β = 1.5, (E) β = 4.0 and (F) β = 10.0.
(TIF)

Figure S4 **Stability zone of the equilibrium point E_1_.** Two-parameter bifurcation diagram to show the stability zone for (A) β = 0.1, (B) β = 0.5, (C) β = 1.0, (D) β = 1.5, (E) β = 4.0 and (F) β = 10.0.
(TIF)

Figure S5 **Stability zone of the inner equilibrium point E_2_.** Two-parameter bifurcation diagram to show the stability zone for (A) β = 0.1, (B) β = 0.5, (C) β = 1.0, (D) β = 1.5, (E) β = 4.0 and (F) β = 10.0.
(TIF)

Figure S6 **Stability zone of the inner equilibrium point E_3_.** Two-parameter bifurcation diagram to show the stability zone for (A) β = 0.1, (B) β = 0.5, (C) β = 1.0, (D) β = 1.5, (E) β = 4.0 and (F) β = 10.0.
(TIF)

Figure S7 **Change of steady state level due to parameter variation for the closure model.** For (A) β = 0.1, (B) β = 1.0, (C) β = 4.0, (D) β = 10.0. Initial value of (p_0_, x, y) = (0.3, 0.01, 0.8).
(TIF)

Figure S8 **Change of steady state level due to change of parameter values for the non-closure model.** Initial value of p = 0.3.
(TIF)

Figure S9 **Change of domain of CV for phytoplankton, for the changes of total nutrient A of the system.** Here half-saturation constant K is kept constant at 1.2 µg N l^−1^. Green lines are the boundary values corresponding to the mean of phytoplankton (p_0_). The dashed line divides the parameter domain for CV<1 and CV>1.
(TIF)

Text S1 **Supplementary text.** This supplementary text contains seven sections (Section S1–S7) explaining model development and analysis referred in the main article.
(PDF)

Dataset S1 **Experimental dataset.** Phytoplankton data as collected by four sampling methods, namely Niskin bottle, Seapoint fluorometer, light emitting diode (LED) sensor and laser sensor, are given in this file.
(XLSX)
