# Supplementary figures and images for: Observations and Models of Highly Intermittent Phytoplankton Distributions
Source: PLoS One. 2014 May 2;9(5):e94797. doi: 10.1371/journal.pone.0094797 (PMC4008380; doi:10.1371/journal.pone.0094797)

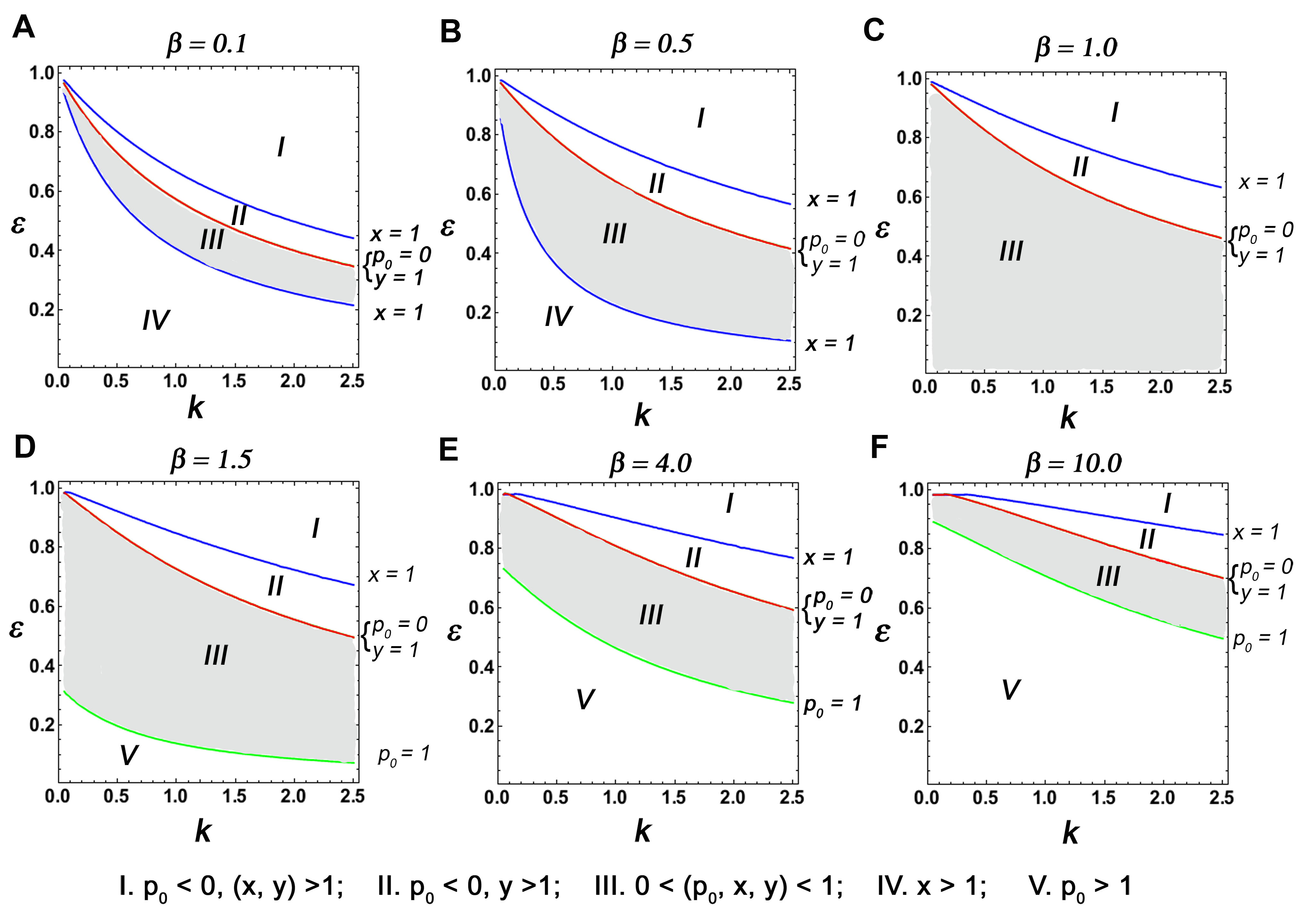

Supplement: Figure S1 — Domain of parameter values for which the equilibrium point E2 exists. Grey shaded regions indicate the domain for (A) β = 0.1, (B) β = 0.5, (C) β = 1.0, (D) β = 1.5, (E) β = 4.0 and (F) β = 10.0. Green, blue and red lines are the boundary values corresponding to the mean of phytoplankton (p0), variance of fluctuating component associated with phytoplankton (x) and nutrient (y) respectively. (TIF) [file pone.0094797.s001.tif]

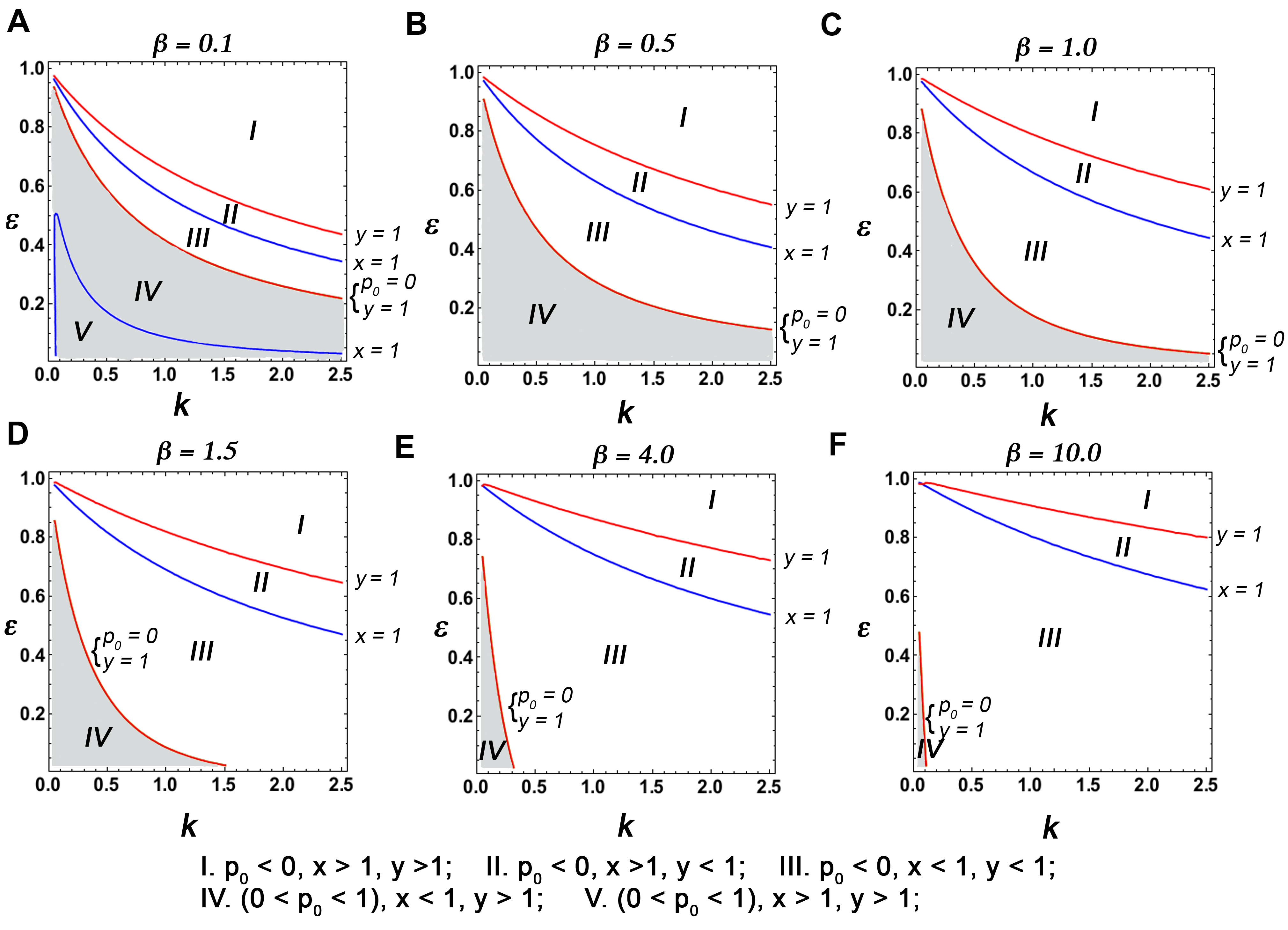

Supplement: Figure S2 — Domain of parameter values for which the equilibrium point E3 exists. Grey shaded regions indicate the domain for (A) β = 0.1, (B) β = 0.5, (C) β = 1.0, (D) β = 1.5, (E) β = 4.0 and (F) β = 10.0. Green, blue and red lines are the boundary values corresponding to the mean of phytoplankton (p0), variance of fluctuating component associated to phytoplankton (x) and nutrient (y) respectively. (TIF) [file pone.0094797.s002.tif]

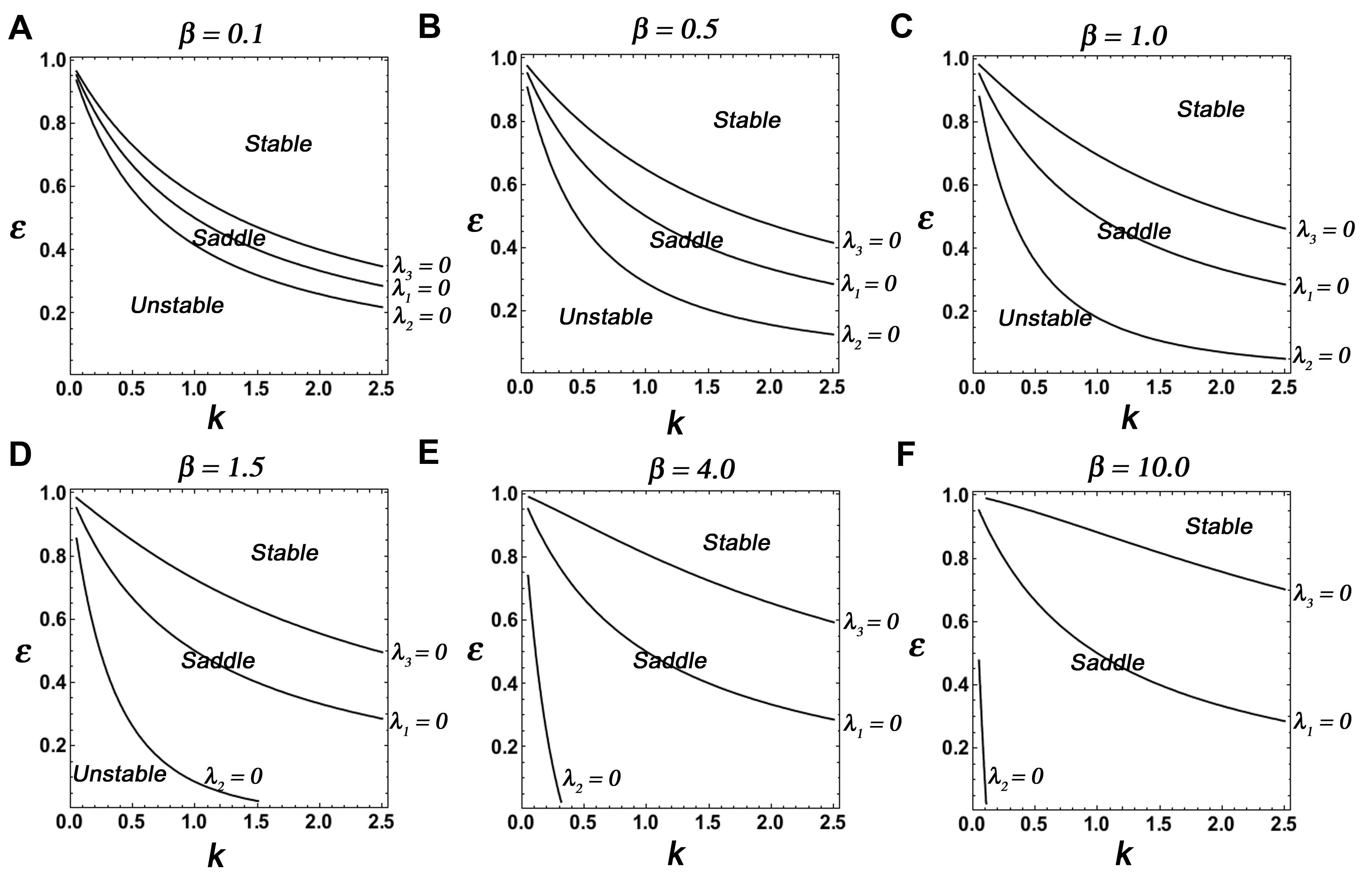

Supplement: Figure S3 — Stability zone of the equilibrium point E0. Two-parameter bifurcation diagram to show the stability zone for (A) β = 0.1, (B) β = 0.5, (C) β = 1.0, (D) β = 1.5, (E) β = 4.0 and (F) β = 10.0. (TIF) [file pone.0094797.s003.tif]

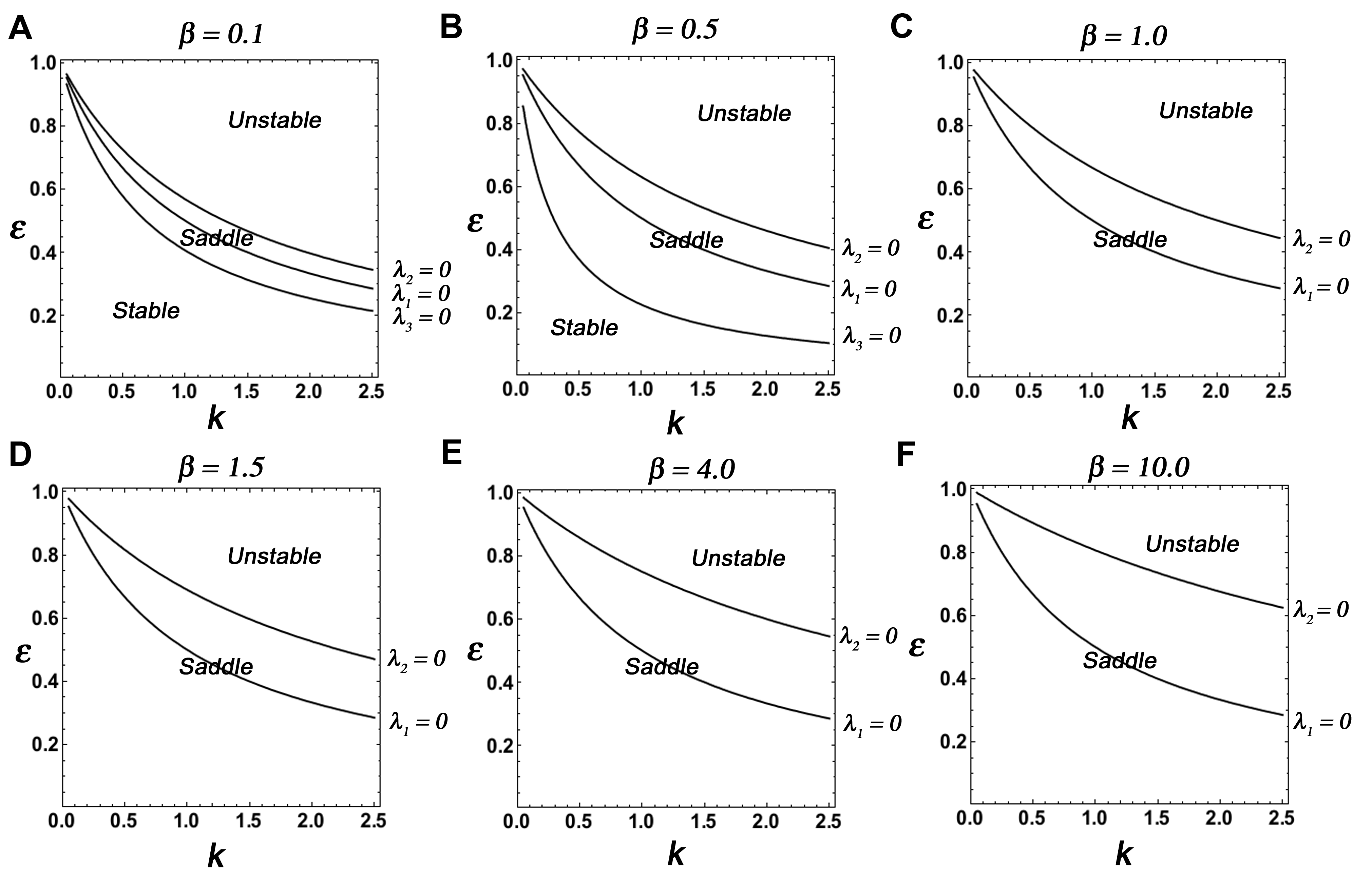

Supplement: Figure S4 — Stability zone of the equilibrium point E1. Two-parameter bifurcation diagram to show the stability zone for (A) β = 0.1, (B) β = 0.5, (C) β = 1.0, (D) β = 1.5, (E) β = 4.0 and (F) β = 10.0. (TIF) [file pone.0094797.s004.tif]

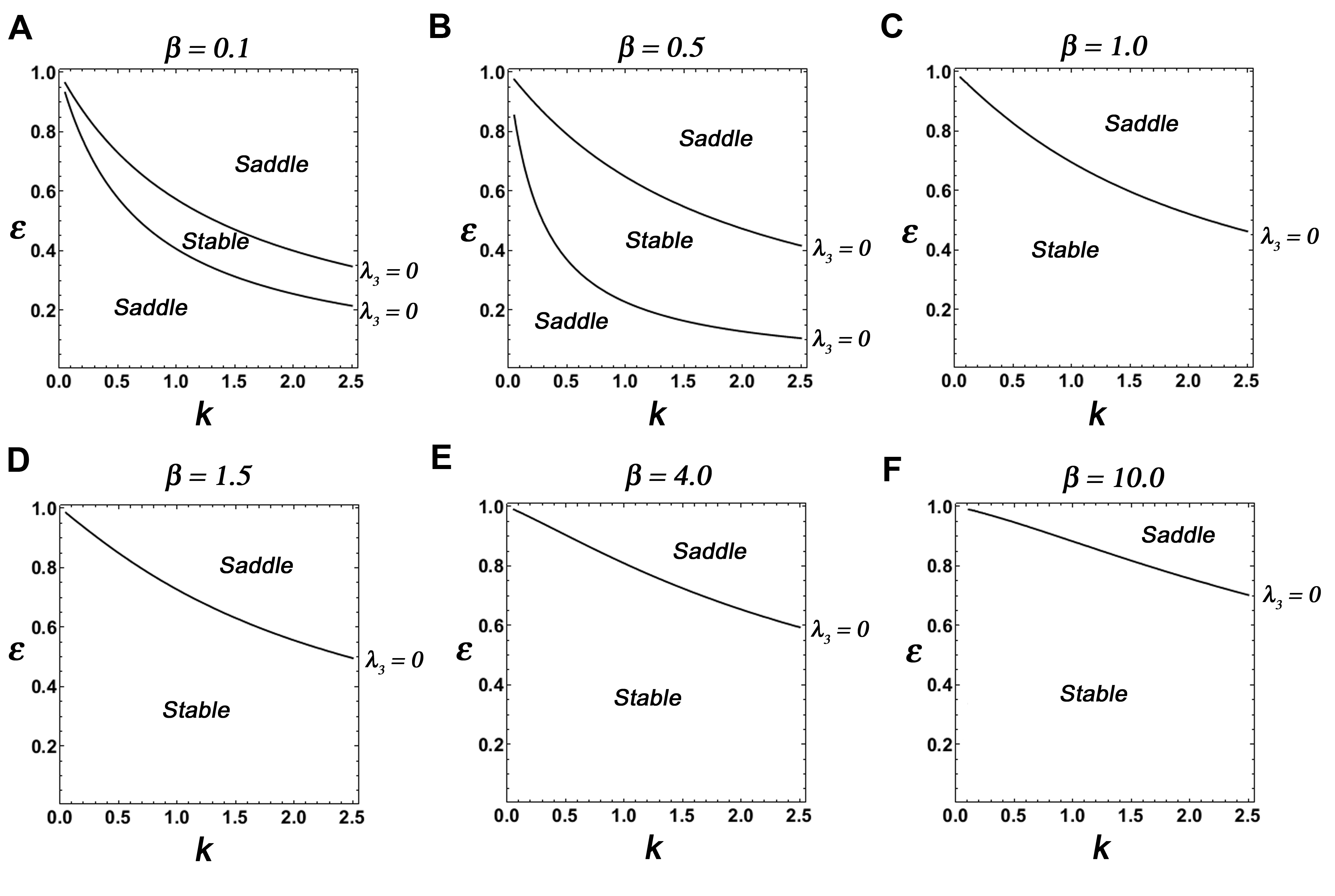

Supplement: Figure S5 — Stability zone of the inner equilibrium point E2. Two-parameter bifurcation diagram to show the stability zone for (A) β = 0.1, (B) β = 0.5, (C) β = 1.0, (D) β = 1.5, (E) β = 4.0 and (F) β = 10.0. (TIF) [file pone.0094797.s005.tif]

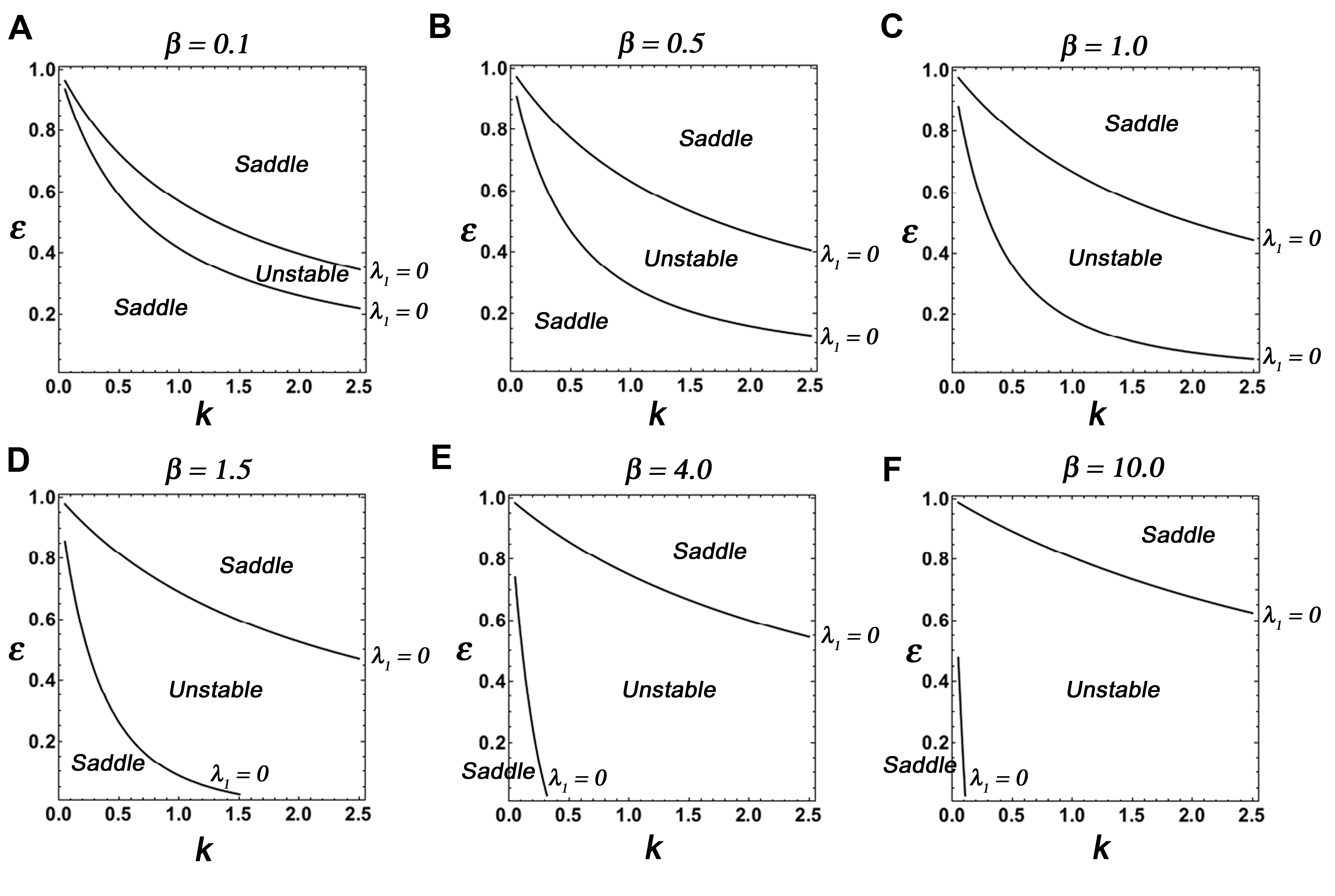

Supplement: Figure S6 — Stability zone of the inner equilibrium point E3. Two-parameter bifurcation diagram to show the stability zone for (A) β = 0.1, (B) β = 0.5, (C) β = 1.0, (D) β = 1.5, (E) β = 4.0 and (F) β = 10.0. (TIF) [file pone.0094797.s006.tif]

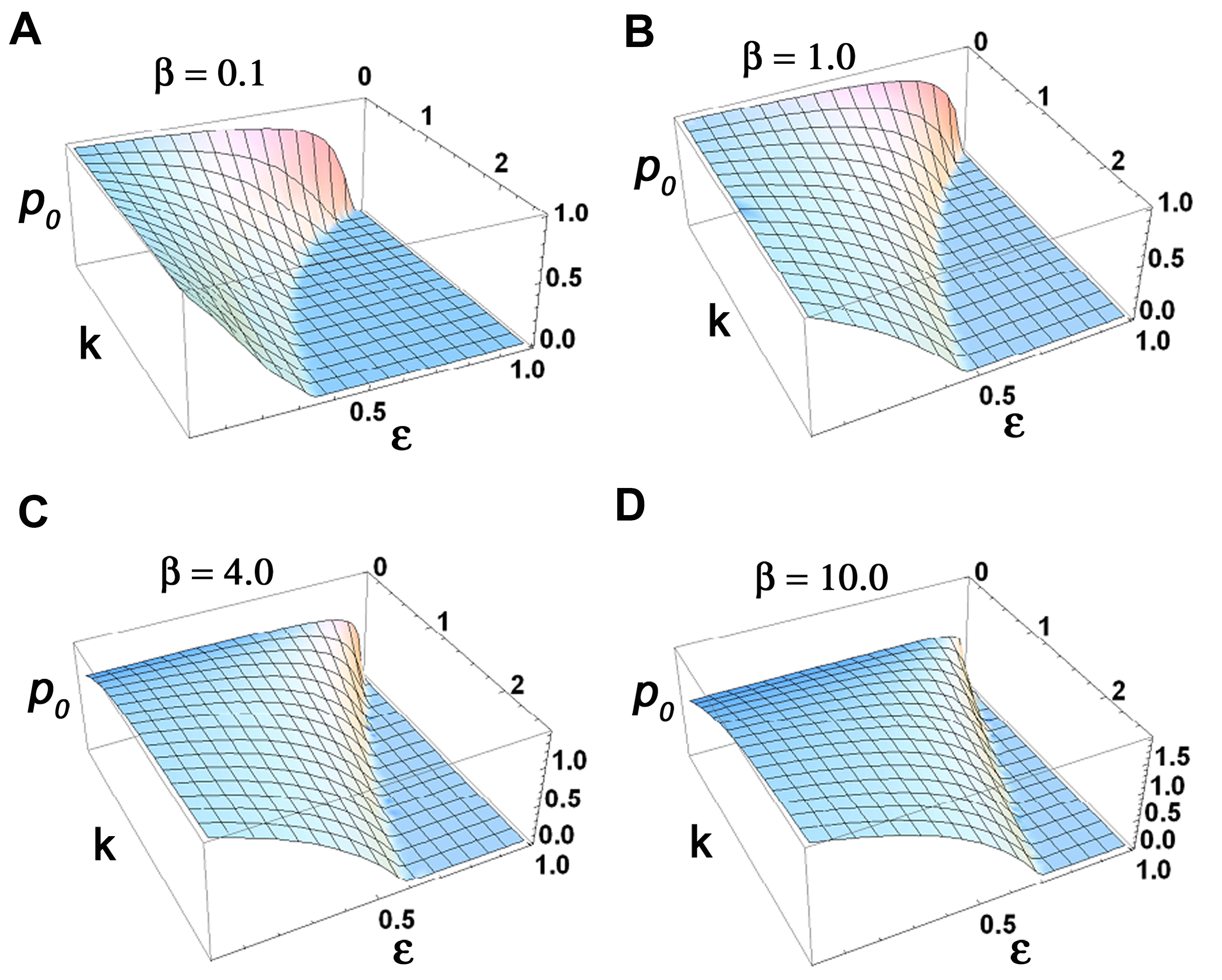

Supplement: Figure S7 — Change of steady state level due to parameter variation for the closure model. For (A) β = 0.1, (B) β = 1.0, (C) β = 4.0, (D) β = 10.0. Initial value of (p0, x, y) = (0.3, 0.01, 0.8). (TIF) [file pone.0094797.s007.tif]

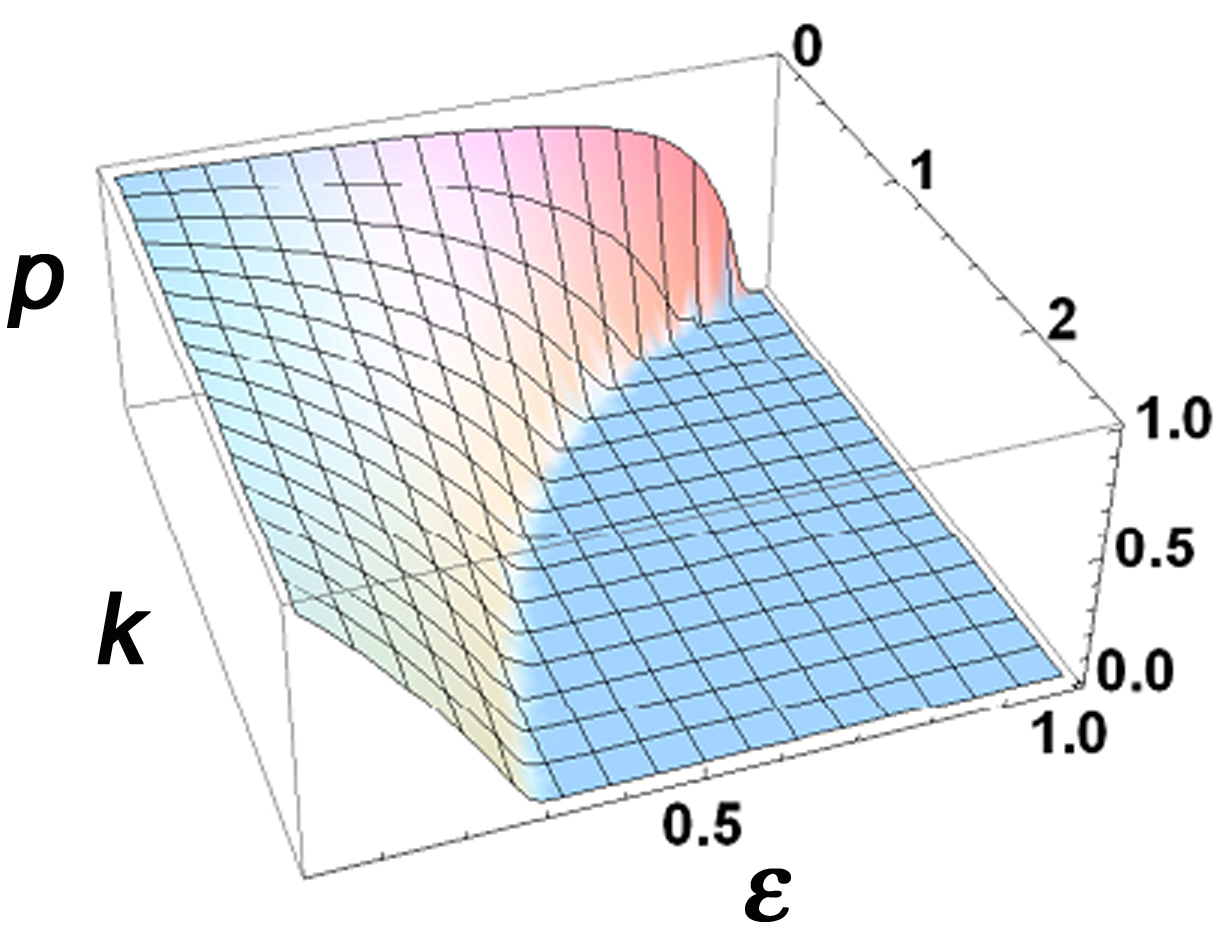

Supplement: Figure S8 — Change of steady state level due to change of parameter values for the non-closure model. Initial value of p = 0.3. (TIF) [file pone.0094797.s008.tif]

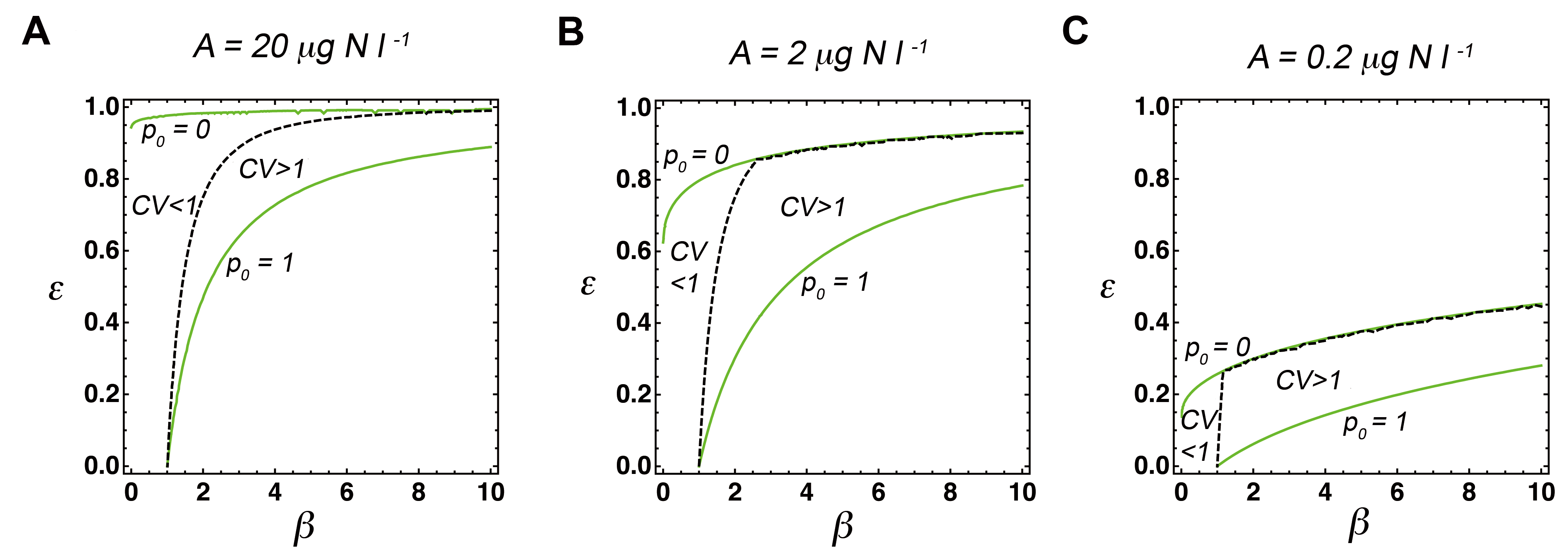

Supplement: Figure S9 — Change of domain of CV for phytoplankton, for the changes of total nutrient A of the system. Here half-saturation constant K is kept constant at 1.2 µg N l−1. Green lines are the boundary values corresponding to the mean of phytoplankton (p0). The dashed line divides the parameter domain for CV<1 and CV>1. (TIF) [file pone.0094797.s009.tif]
