# Supplementary material for: Observations and Models of Highly Intermittent Phytoplankton Distributions
Source: PLoS One. 2014 May 2;9(5):e94797. doi: 10.1371/journal.pone.0094797 (PMC4008380; doi:10.1371/journal.pone.0094797)
Supplement: Text S1 — Supplementary text. This supplementary text contains seven sections (Section S1–S7) explaining model development and analysis referred in the main article. (PDF) [file pone.0094797.s010.pdf]

Text S1

Observations and models of highly intermittent phytoplankton distributions

Sandip Mandal, Christopher Locke, Mamoru Tanaka, Hidekatsu Yamazaki

---

Table of contents

S1. NPZ model……………………………………………………………………………...1
S2. Model development using the closure approach ………………………..……………….2
S3. Equilibrium points…………………………………………………………………......3
S4. Domain of parameter values for the equilibrium points……………………………………..5
S5. Stability condition……………………………………………………………………………6
S6. Steady state dynamics for the variation of parameters……………………………………..12
S7. Coefficient of variation for phytoplankton…………………………………………………14

S1. NPZ Model

In ocean ecology, basic ecosystem models are developed using three state variables: nutrient (*N*), phytoplankton (*P*) and zooplankton (*Z*). Nitrogen content in each variable is used as the currency for these systems, as nitrogen often limits primary production in the ocean. The time evolution equations for the most general NPZ system can be written as:

$$\frac{dP}{dt} = g(I)u(N)\,P - h(P)\,Z - d(P)\,P$$

$$\frac{dZ}{dt} = \phi\, h(P)\,Z - m(Z)\,Z$$

$$\frac{dN}{dt} = -g(I)u(N)\,P + (1-\phi)h(P)\,Z + d(P)\,P + m(Z)Z$$

The basic model varies by the choice of five transfer functions of the system: *g(I), u(N)*, *h(P)*, *d(P)* and *m(Z)*. Here *g(I)* represents the phytoplankton growth response to light with intensity *I*, *u(N)* represents phytoplankton nutrient uptake, *h(P*) is the zooplankton grazing rate, and *d(P)* and *m(Z)* are the loss terms due to death of phytoplankton and zooplankton respectively. The term ϕ represents zooplankton assimilation. Different functional forms have been chosen by various modellers to explain different situations.

As the aim of this paper is to develop a new methodology of ocean ecosystem modelling and to study the effect of spatial fluctuation of the model variables on the system dynamics, at the initial stage, we concentrate only on *N* and *P* variables and choose a simple form for the transfer functions. Choosing *g(I)* and *d(P)* to be constant values *C* and *D* respectively and

choosing *u(N)* to follow a saturating response (Michaelis-Menten uptake of dissolved nutrient), the NP model for temporal variation of *N* and *P* variable looks as follows:

$$\frac{dP}{dt} = C\,\frac{N}{K+N}\,P - D\,P$$

$$\frac{dN}{dt} = -C\,\frac{N}{K+N}\,P + D\,P$$

K is the nutrient uptake half saturation constant.

S2. Model development using the closure approach

The general form of equations we would like to solve for *N* variables is:

$$\frac{dX_i}{dt} = F_i(X_i)\,.$$

Reynolds decomposition is done by then decomposing the variable X_i into a mean and fluctuating part:

$$X_i = \overline{X}_i + X_i'\,.$$

This is then substituted into the above equation of motion and Taylor expanded to give:

$$\frac{d}{dt}(\overline{X}_i + X_i') = \sum_{n=0}^{\infty}\frac{1}{n!}\hat{D}^n F_i(\overline{X}_i) = e^{\hat{D}} F_i(\overline{X}_i)\,,$$

where we have defined the differential operator $\hat{D}$, which generates the Taylor expansion as:

$$\hat{D} = \sum_{i=1}^{N} X_i'\,\frac{\partial}{\partial X_i}\,.$$

Taking the ensemble average of the equation of state gives:

$$\frac{d\overline{X}_i}{dt} = < e^{\hat{D}} > F_i(\overline{X}_i)\,.$$

Subtracting this from the equation of state gives an equation governing the fluctuating part:

$$\frac{dX_i'}{dt} = (\,e^{\hat{D}} - < e^{\hat{D}} >\,)\,F_i(\overline{X}_i)\,.$$

Finally, the time derivatives of the second order covariance terms are:

$$\frac{d}{dt} < X_i' X_j' > = < \frac{dX_i'}{dt} X_j' + \frac{dX_j'}{dt} X_i' > = < X_j' e^{\hat{D}} F_i + X_i' e^{\hat{D}} F_j >\,.$$

From the form of the equation here, it should be clear how time derivatives of any order terms can be written by using the product rule of derivatives and substituting in $e^{\hat{D}} F_i$ for derivatives of fluctuating terms.

Assuming that any solution to the equations of state is analytic, the above equations are exact. For what follows, we will only deal with terms up to order 2 in powers of fluctuating variables. In formulating these equations, it is assumed that the X_i' variables follow joint lognormal probability distribution function, which makes it possible for the third order term to vanish, and also we ignored the fourth and higher order terms in this analysis.

S3. Equilibrium points

Equilibrium/steady state points are the values of the state variables at which the source and loss rates are exactly balanced, i.e. the time variation becomes zero. The system of equations for the closure model (See equations 11–13 in main text) possesses the following six equilibrium points (p_0*, x*, y*),

$$E_0 = (0,0,1) \tag{1}$$

$$E_1 = \left(\left(1-\frac{k\varepsilon}{1-\varepsilon}\right), 1, 0\right) \tag{2}$$

and $$E_{1+i} = \left(p_{0i}*, \frac{kp_{0i}*^2}{\beta(k+1-p_{0i}*)(1-\varepsilon)}, \frac{(k+1-p_{0i}*)(k-(k+1-p_{0i}*)(1-\varepsilon))^2}{\beta(k+1-p_{0i}*)(1-\varepsilon)}\right). \tag{3}$$

The expression for p_{0i}* (i = 1, 2, 3, 4) is obtained by solving a fourth order equation and the possible solutions are as follows:

$$p_{01}* = 1+k-\frac{1}{2}\sqrt{\gamma+\delta}+\frac{1}{2}\sqrt{-\gamma+2\delta+\frac{2k\beta}{(1-\varepsilon)\sqrt{\gamma+\delta}}} \tag{4}$$

$$p_{02}* = 1+k-\frac{1}{2}\sqrt{\gamma+\delta}-\frac{1}{2}\sqrt{-\gamma+2\delta+\frac{2k\beta}{(1-\varepsilon)\sqrt{\gamma+\delta}}} \tag{5}$$

$$p_{03}* = 1+k+\frac{1}{2}\sqrt{\gamma+\delta}+\frac{1}{2}\sqrt{-\gamma+2\delta-\frac{2k\beta}{(1-\varepsilon)\sqrt{\gamma+\delta}}} \tag{6}$$

$$p_{04}* = 1+k+\frac{1}{2}\sqrt{\gamma+\delta}-\frac{1}{2}\sqrt{-\gamma+2\delta-\frac{2k\beta}{(1-\varepsilon)\sqrt{\gamma+\delta}}} \tag{7}$$

with, $$\delta = \frac{4k(1+k)}{3(1-\varepsilon)} \tag{8}$$

$$\gamma = \frac{16 \times 2^{\frac{1}{3}}\, k^2 (1+k)^2}{3\,\eta^{\frac{1}{3}}} + \frac{\eta^{\frac{1}{3}}}{3 \times 2^{\frac{1}{3}}\,(1-\varepsilon)^2} \qquad (9)$$

and

$$\eta = \sqrt{\left(128 k^3 (1+k)^3 (1-\varepsilon)^3 + 27 k^2 \beta^2 (1-\varepsilon)^4\right)^2 - 16384 k^6 (1+k)^6 (1-\varepsilon)^6} + 128\, k^3 (1+k)^3 (1-\varepsilon)^3 + 27 k^2 \beta^2 (1-\varepsilon)^4 \;. \qquad (10)$$

E_{i+1} exist for $\beta > 0$, $\varepsilon < 1$ and $-\gamma + 2\delta \pm \frac{2k\beta}{(1-\varepsilon)\sqrt{\gamma+\delta}} \geq 0$. But within our domain of parameters (See table 2 in main text) $-\gamma + 2\delta - \frac{2k\beta}{(1-\varepsilon)\sqrt{\gamma+\delta}} < 0$. This implies p_{03} and p_{04} are complex, and therefore E_4 and E_5 are not feasible solutions.

Among four possible values of p_{0i}*, only two, (p_{01} and p_{02}), are ecologically feasible, which gives two feasible equilibrium points. Between these two points, one (say E_2) corresponds to the condition when the covariance of the fluctuating components $< N'P' >$ is positive, and in this case, all the scaled variables (p_0*, x*, y*) lie between 0 and 1. The other equilibrium point (say E_3) corresponds to the condition when the covariance of the fluctuating components $< N'P' >$ is negative, and in this case, p_0* lies between 0 and 1. However, x* and y* can exceed the value 1.

Depending on the sign of covariance term $< N'P' >$, the inner equilibrium point E_2 or E_3 can exist. So there are three possible equilibrium points for the NP closure model, among which two (E_0, E_1) are at the boundary and the other one is the inner equilibrium point (either E_2 or E_3).

There exist only two equilibrium points for the original model (equation 15 in main text), which are

$$p_1{}^* = 0 \qquad (11)$$

or

$$p_2{}^* = \left(1 - \frac{k\,\varepsilon}{1-\varepsilon}\right) \qquad (12)$$

with the condition ε < 1.

The equilibrium point E_0 of the closure model and p_1* of the non-closure model imply that there is no phytoplankton present in the system. In this case, for the closure model, the variance of nutrient reaches to 1 and variance of phytoplankton is 0.

Both of the variables N and P are present in the system for the equilibrium point E_1 of the closure model and p_2* of the non-closure model. In this case, for the closure model, the variance of phytoplankton reaches to 1 and the variance of nutrient is 0.

S4. Domain of parameter values for the equilibrium points

The inner equilibrium point for the closure model, either E_2 (when $< N'P' >$ is positive) or E_3 (when $< N'P' >$ is negative), is the most general case, where both variables are present along with their respective variance. The domains of parameter space for which the equilibrium points E_2 and E_3 exist for different β values are shown in Figure S1 and Figure S2 respectively. According to our assumptions, p_0 should lie within 0 and 1. The value of x and y also lies between 0 and 1 for the equilibrium point E_2, but for the equilibrium point E_3 the value of x and y can exceed 1. Here we should remember that there is no such realistic situation where the nutrient content of the system is zero and phytoplankton is at maximum (i.e. 1 for the dimensionless system).

Figure S1 A shows that for $\beta = 0.1$ the equilibrium point E_2 exhibits feasible value for a narrow k-ε parameter space. As the value of β increases, the parameter domain for E_2 increases up to a certain value of β (Figure S1 A-C), and then for further increase in β this area decreases (Figure S1 D-F). The value of the parameter β changes according to the magnitude of spatial variation of the system.

The domain of parameters for existence of the equilibrium point E_3 is shown in Figure S2. This figure shows that for $\beta = 0.1$ the equilibrium point E_3 exhibits feasible value for a certain k-ε parameter space (region IV and V in Figure S2). As the value of β increases, the parameter domain for E_3 decreases. Here we should remember that the equilibrium point E_3 exists only when covariance of fluctuating terms is negative.

S5. Stability condition

We investigate the local asymptotic stability of the closure system (11–13 in main text) around the equilibrium points E_0, E_1, E_2 and E_3. The stability condition of the equilibrium points is based on how the system behaves when variables are perturbed slightly from the equilibrium values. If the variables return to their steady state, then they are stable, or otherwise, if they diverge from that point, they are unstable. This qualitative behaviour of the equilibrium point is found by linearizing the system around the equilibrium point E^* and writing the sets of equations as:

$$\frac{dX_i}{dt} = J(E^*)\, X_i + \text{Nonlinear terms}, \qquad (13),$$

where X_i is the set of variables and J is called Jacobian matrix or community matrix at E^*, whose elements are partial derivatives of the model equations with respect to their variables. The approximated solution of equation (13) is the sum of exponential terms, where the exponents equal to the real parts of eigenvalues of the Jacobian matrix J. Therefore, the sign of real parts of eigenvalues determines the nature of stability in the vicinity of E^*.

The expression for Jacobian matrix J for the set of equations (11–13 in main text) at the equilibrium point E^* is given by

$$J(E^*) = \left. \begin{pmatrix} \frac{\partial}{\partial p_0}\left(\frac{\partial p_0}{\partial \tau}\right) & \frac{\partial}{\partial x}\left(\frac{\partial p_0}{\partial \tau}\right) & \frac{\partial}{\partial y}\left(\frac{\partial p_0}{\partial \tau}\right) \\ \frac{\partial}{\partial p_0}\left(\frac{\partial x}{\partial \tau}\right) & \frac{\partial}{\partial x}\left(\frac{\partial x}{\partial \tau}\right) & \frac{\partial}{\partial y}\left(\frac{\partial x}{\partial \tau}\right) \\ \frac{\partial}{\partial p_0}\left(\frac{\partial y}{\partial \tau}\right) & \frac{\partial}{\partial x}\left(\frac{\partial y}{\partial \tau}\right) & \frac{\partial}{\partial y}\left(\frac{\partial y}{\partial \tau}\right) \end{pmatrix} \right|_{E^*} = \begin{pmatrix} A_{11} & A_{12} & A_{13} \\ A_{21} & A_{22} & A_{23} \\ A_{31} & A_{32} & A_{33} \end{pmatrix}.$$

where

$$A_{11} = \frac{1}{1+k-p_0*} + \frac{p_0*^2}{(1+k-p_0*)^2} + \frac{k(1-x*-2y*)\beta}{(1+k-p_0*)^3} - \frac{p_0*\{(1+k-p_0*)^2 + 2k(1+k-p_0*)^2 + 3k\,y*\beta\}}{(1+k-p_0*)^4} - \varepsilon,$$

$$A_{12} = -\frac{k\beta}{2(1+k-p_0*)^2},$$

$$A_{13} = -\frac{k(1+k+p_0*)\beta}{2(1+k-p_0*)^3},$$

$$A_{21} = \frac{k\{1-3x*-y*+p_0*(1+x*-y*)+k(1-3x*-y*)\}}{(1+k-p_0*)^3},$$

$$A_{22} = \frac{2(1-p_0*)}{1+k-p_0*} - \frac{k\,p_0*}{(1+k-p_0*)^2} - 2\varepsilon,$$

$$A_{23} = -\frac{k\,p_0*}{(1+k-p_0*)^2},$$

$$A_{31} = \frac{k\{1-x*-3y*-p_0*(1-x*+y*)+k(1-x*-3y*)\}}{(1+k-p_0*)^3},$$

$$A_{32} = \frac{1-p_0*}{1+k-p_0*} - \varepsilon,$$

$$A_{33} = \frac{1-p_0*}{1+k-p_0*} - \frac{2k\,p_0*}{(1+k-p_0*)^2} - \varepsilon.$$

Eigenvalues (λ) of this Jacobian matrix can be obtained by solving the characteristic equation

$J(E^*) - I\lambda = 0$, where $I = \begin{pmatrix} 1 & 0 & 0 \\ 0 & 1 & 0 \\ 0 & 0 & 1 \end{pmatrix}$.

a. Stability condition for the system around E_0

The eigenvalues of the Jacobian matrix J at equilibrium point E_0 can be written as follows:

$$\lambda_1 = \frac{2}{1+k} - 2\epsilon$$

$$\lambda_2 = \frac{2 + k^2(2-6\epsilon) - 2\epsilon - 2k^3\epsilon + k(4-\beta-6\epsilon) - k\sqrt{\beta(4+8k+4k^2+\beta)}}{2(1+k)^3}$$

$$\lambda_3 = \frac{2 + k^2(2-6\epsilon) - 2\epsilon - 2k^3\epsilon + k(4-\beta-6\epsilon) + k\sqrt{\beta\,(4+8k+4k^2+\beta)}}{2(1+k)^3}$$

For any real positive parameter values, all the eigenvalues are real. Therefore, only steady state bifurcation can occur, and there is no possibility of oscillation of the system around this equilibrium point. A two-parameter bifurcation diagram is shown in Figure S3 for six different β values.

An equilibrium point is stable in a region where the real eigenvalues or real parts of complex eigenvalues are negative. If these are all positive, then the equilibrium point is unstable, and if there are some eigenvalues with positive real part and the rest are negative, then the equilibrium point is in the saddle zone. Figure S3 demonstrates a region of space where the equilibrium point E_0 is stable/saddle/unstable for β = 0.1, 0.5, 1.0, 1.5, 4.0 and 10.0. Here the dimensionless parameter, ε, which is the ratio of death rate to the maximum growth rate of phytoplankton, is varied from 0.025 to 0.99 and the nutrient uptake half saturation constant k value is varied from 0.05 to 2.5. From these figures we see that as the β value increases, the parameter domain for which E_0 is stable reduces.

b. Stability condition for the system around E_1

The eigenvalues of the Jacobi matrix J at equilibrium point E_1 can be written as follows:

$$\lambda_1 = \frac{2(1-\epsilon)(-1+\epsilon+k\epsilon)}{k}$$

$$\lambda_2 = \frac{-k(1-\epsilon)^2 + k^2(1-\epsilon)\epsilon - \sqrt{k^2\beta(1-\epsilon)^4}}{k^2}$$

$$\lambda_3 = \frac{-k(1-\epsilon)^2 + k^2(1-\epsilon)\epsilon + \sqrt{k^2\beta(1-\epsilon)^4}}{k^2}$$

because $\varepsilon < 1$; for any real positive parameter values, all the eigenvalues are real. Therefore, only steady state bifurcation can occur, and similar to the equilibrium point E_0, there is no possibility of oscillation of the system around this equilibrium point (E_1). The two-parameter bifurcation diagram for this point is shown in Figure S4 for six different β values. This figure depicts the

stability region of equilibrium point E_1 for β = 0.1, 0.5, 1.0, 1.5, 4.0 and 10.0 in the parameter domain of k (0.05 to 2.5) and ε (0.025 to 0.99). For low β values this equilibrium point is stable when both k and ε are low, but for high k and high ε values this point becomes unstable. As β increases, the stability region in the parameter space decreases. For β = 1.0 and above, this point is not stable at all in the above mentioned parameter space.

c. Stability condition for the system around inner equilibrium point E_2

To study the qualitative behaviour of the system neighbouring the equilibrium point E_2, we have plotted a two-parameter bifurcation diagram at this point as well. In our studied parameter domain of k (0.05 to 2.5) and ε (0.025 to 0.99), two among three eigenvalues, λ_1 and λ_2, are always negative. Figure S5 depicts the condition for the third eigenvalue (λ_3) to be zero in k-ε parameter space for six β values. From Figure S5 A it is observed that for β = 0.1, the equilibrium point E_2 is stable in a narrow region of parameter space where the eigenvalue λ_3 is negative, and as β value increases, this domain of stability increases as shown in Figure S5 B-F.

Comparing this figure with Figure S1, it can be stated that the domain of parameters in which the equilibrium point E_2 is ecologically feasible is also the domain of stability for this point, or in other words, we can say that the inner-equilibrium point E_2 is stable in the entire region where it is ecologically feasible.

Comparing the stability zones of the three equilibrium points E_0, E_1 and E_2 in Figures S3, S4 and S5, one can easily state that in the region of parameter space where the equilibrium point E_0 (or E_1, or E_2) is stable, the other two points are not stable for any β values.

d. Stability condition for the system around inner equilibrium point E_3

This equilibrium point E_3 exists only when the covariance term between fluctuating components is negative. In our studied parameter domain of k (0.05 to 2.5) and ε (0.025 to 0.99), two eigenvalues of the Jacobian matrix J (λ_2 and λ_3) at this equilibrium point are always positive. Therefore, the equilibrium point E_3 is never stable in this region of space. Stability zone of this equilibrium point is shown in Figure S6.

From our analysis we have observed that the equilibrium points E_0, E_1 and E_2 are stable for a specific range of parameters but that these three points are never stable simultaneously. The domain of parameters in which the equilibrium point E_2 is ecologically feasible is also the domain of stability for this point, or in other words, we can say that the inner-equilibrium point E_2 is stable in the entire region where it is ecologically feasible. We have also observed that the increase of β value increases the stability zone of the system around E_2. The equilibrium point E_3 exists only for negative covariance, but it is never stable in our studied parameter domain of k (0.05 to 2.5) and ε (0.025 to 0.99). Therefore, we concentrate only on the inner equilibrium point E_2 for our further analysis.

S6. Steady state dynamics for the variation of parameters

In earlier sections we looked at the parameter domain of the NP closure model where the equilibrium points are stable. In this section we will look at the steady state dynamics of the

model variable p_0 with variation of parameters for different β values. Here, in the dimensionless frame, we have varied *k*-value from 0.05 to 2.5 and *ε*-value from 0.025 to 0.99.

Figure S7 A-D shows the change of the steady state level of p_0 for $\beta = 0.1, 1.0, 4.0$ and $\beta = 10.0$ respectively. Each point of this surface represents the steady state value of p_0 corresponding to the value of k and *ε*. The initial values for this simulation were chosen to be ($p_0(0)$, $x(0)$, $y(0)$) = (*0.3, 0.01, 0.8*). These figures show that depending on the choice of parameter combinations, the steady state level of p_0 can vary from zero to its boundary value, i.e., 1. Also, by comparing these Figures (S7 A-D), we can say that as the *β* value increases, the domain for a nonzero steady state value of p_0 increases. This ecologically implies that if the variance of overall fluctuations (nutrient and phytoplankton) increases, the parameter domain for phytoplankton would also increase in the system. In other words, the stability zone of phytoplankton increases with the increase of spatial fluctuations.

For zero fluctuation, the value of *β* becomes zero. In this situation the equation for the mean value of phytoplankton (p_0) of the closure model (equation 11 in main text) approaches the equation for phytoplankton (*p*) of the non-closure model (equation 15 in main text). Figure S8 is a plot of the steady state value of phytoplankton in such a situation when $\beta = 0$. Here the parameter domain, for not having phytoplankton in the system, is the maximum in comparison to the cases for nonzero *β* value.

Comparing Figure S7 and S8, one can state that there are some parameter combinations where the steady state value for the non-closure model and the closure model coincides even for nonzero *β* value. In the results section of main text we have described this rigorously.

S7. Coefficient of variation for phytoplankton

Coefficient of variation of a random variable is defined as the ratio of standard deviation and mean of that variable. Its value can be greater or less than 1. Figure S9 shows the domain of coefficient of variation (CV) for phytoplankton, as obtained from the closure model, in *ε*-*β* parameter space. The change of parameter domain for the CV for phytoplankton is observed for three different *A* values. This is observed that as the total nutrient of the system *A* decreases, the relative parameter domain for CV < 1 decreases with respect to the parameter domain for CV > 1.
